# Supplementary figures and images for: Bioinformatics analyses and experimental validation of ferroptosis-related genes in bronchopulmonary dysplasia pathogenesis
Source: PLoS One. 2024 Jun 14;19(6):e0291583. doi: 10.1371/journal.pone.0291583 (PMC11178182; doi:10.1371/journal.pone.0291583)

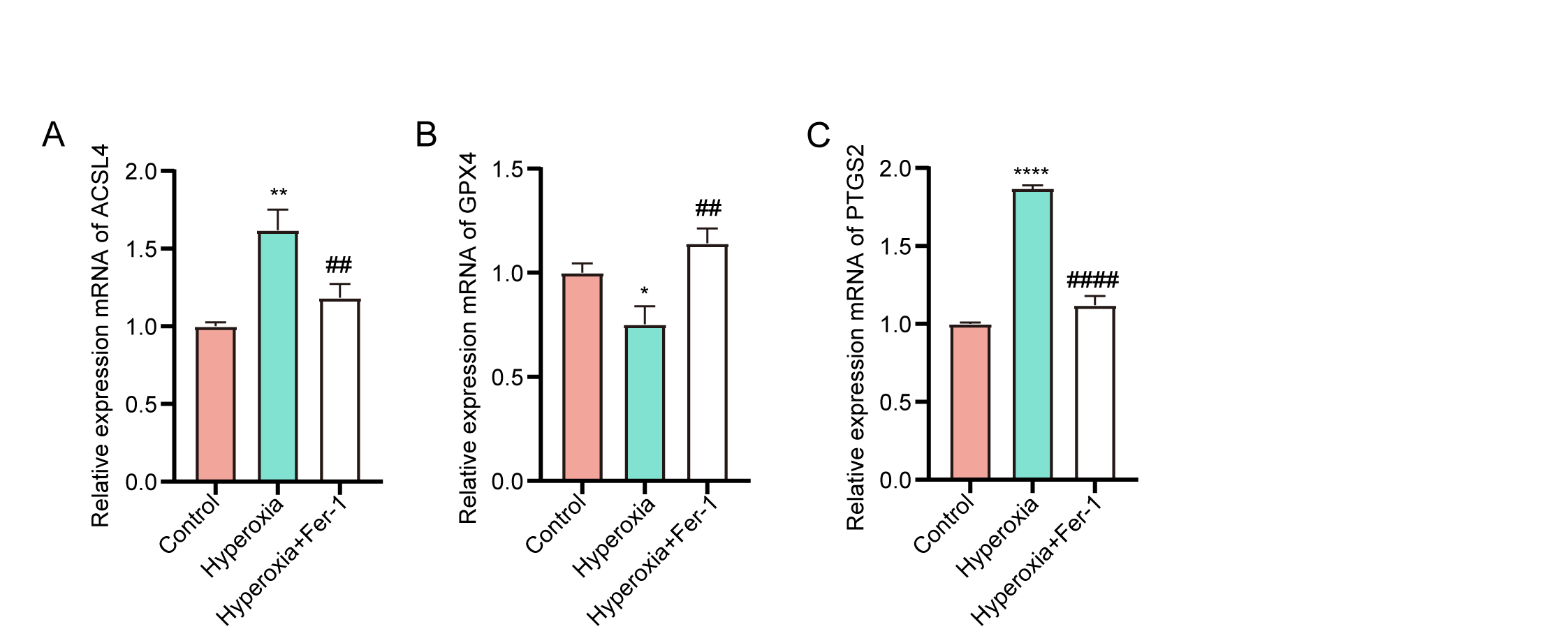

Supplement: S1 Fig — (A) ACSL4. (B) GPX4. (C) PTGS2. * P < 0.05, ** P < 0.01, **** P < 0.0001 compared with the control group. ## P < 0.01, #### P < 0.0001 compared with hyperoxia group. (TIF) [file pone.0291583.s001.tif]

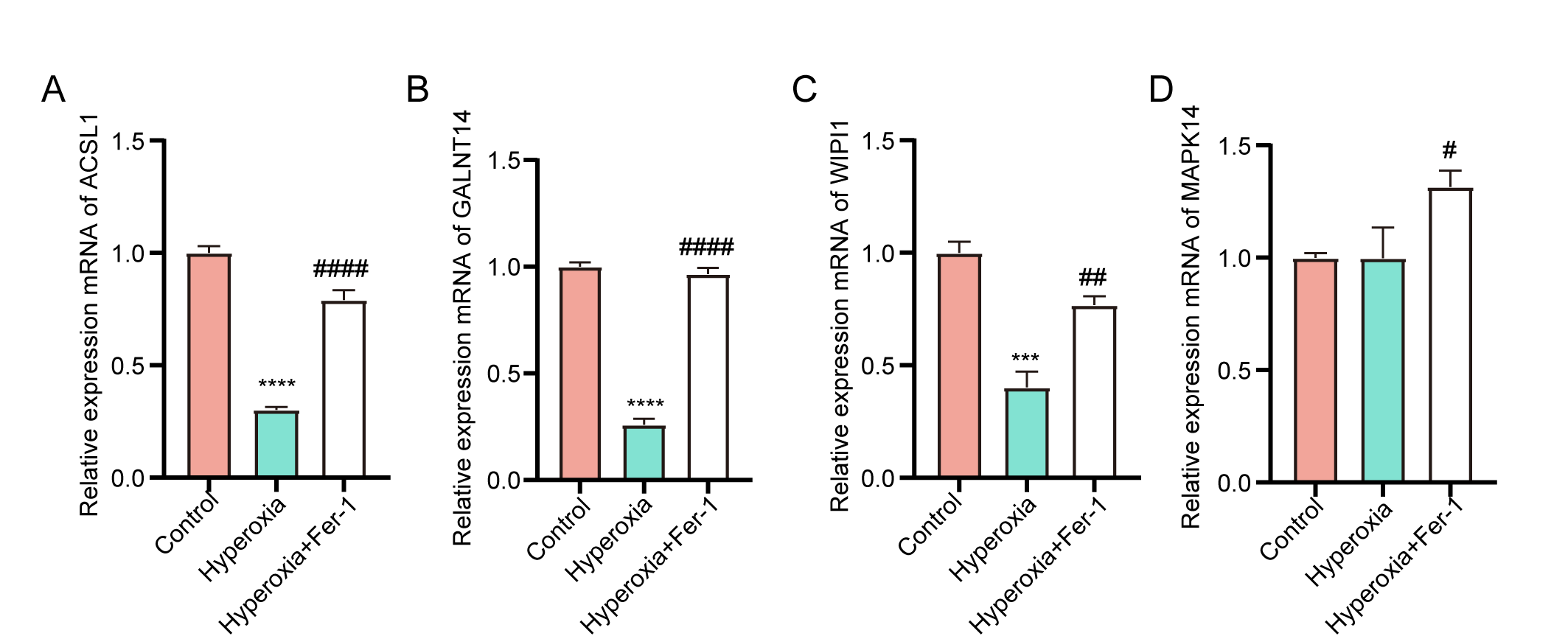

Supplement: S2 Fig — (A) ACSL1. (B) GALNT14. (C) WIPI1. (D) MAPK14. ***P < 0.001, ***P < 0.001 compared with the control group. # P < 0.05, ## P < 0.01, #### P < 0.0001 compared with hyperoxia group. (TIF) [file pone.0291583.s002.tif]

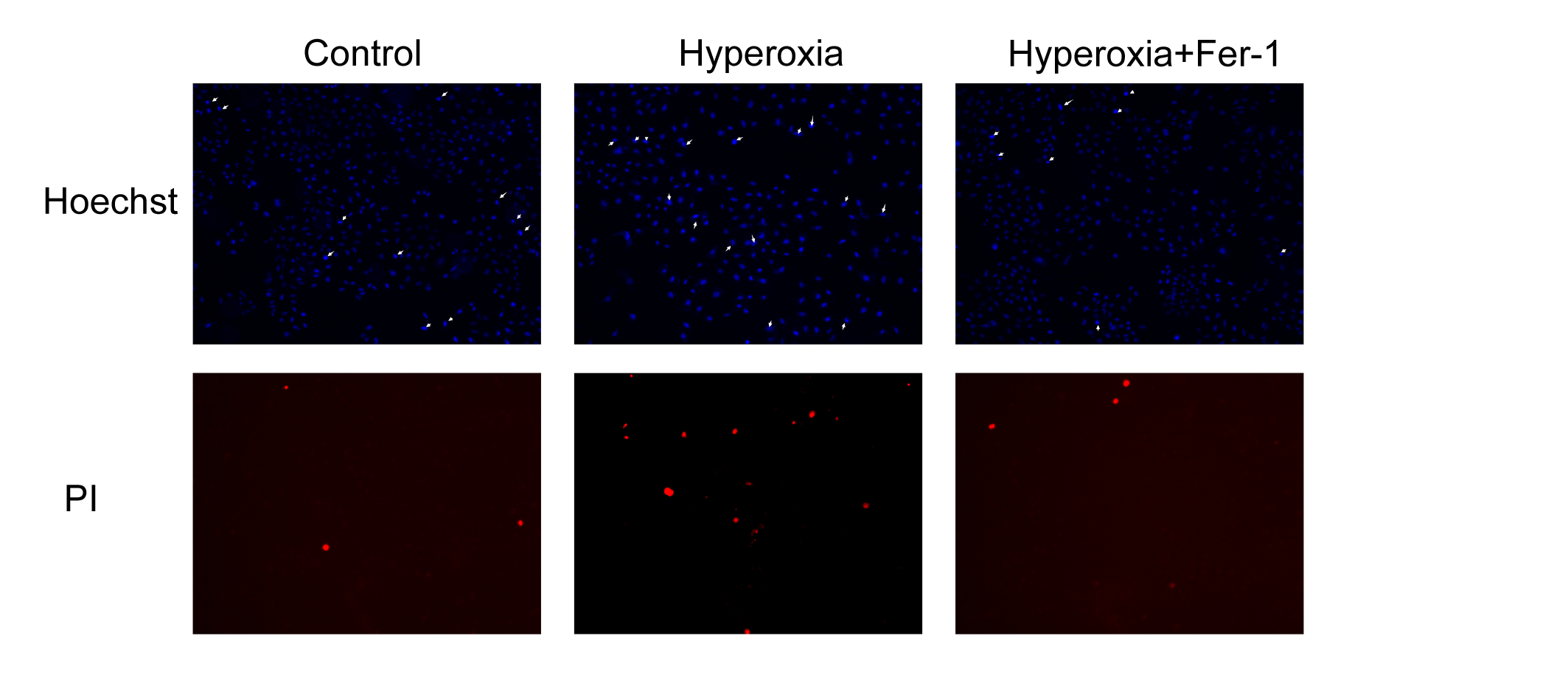

Supplement: S3 Fig — (TIF) [file pone.0291583.s003.tif]

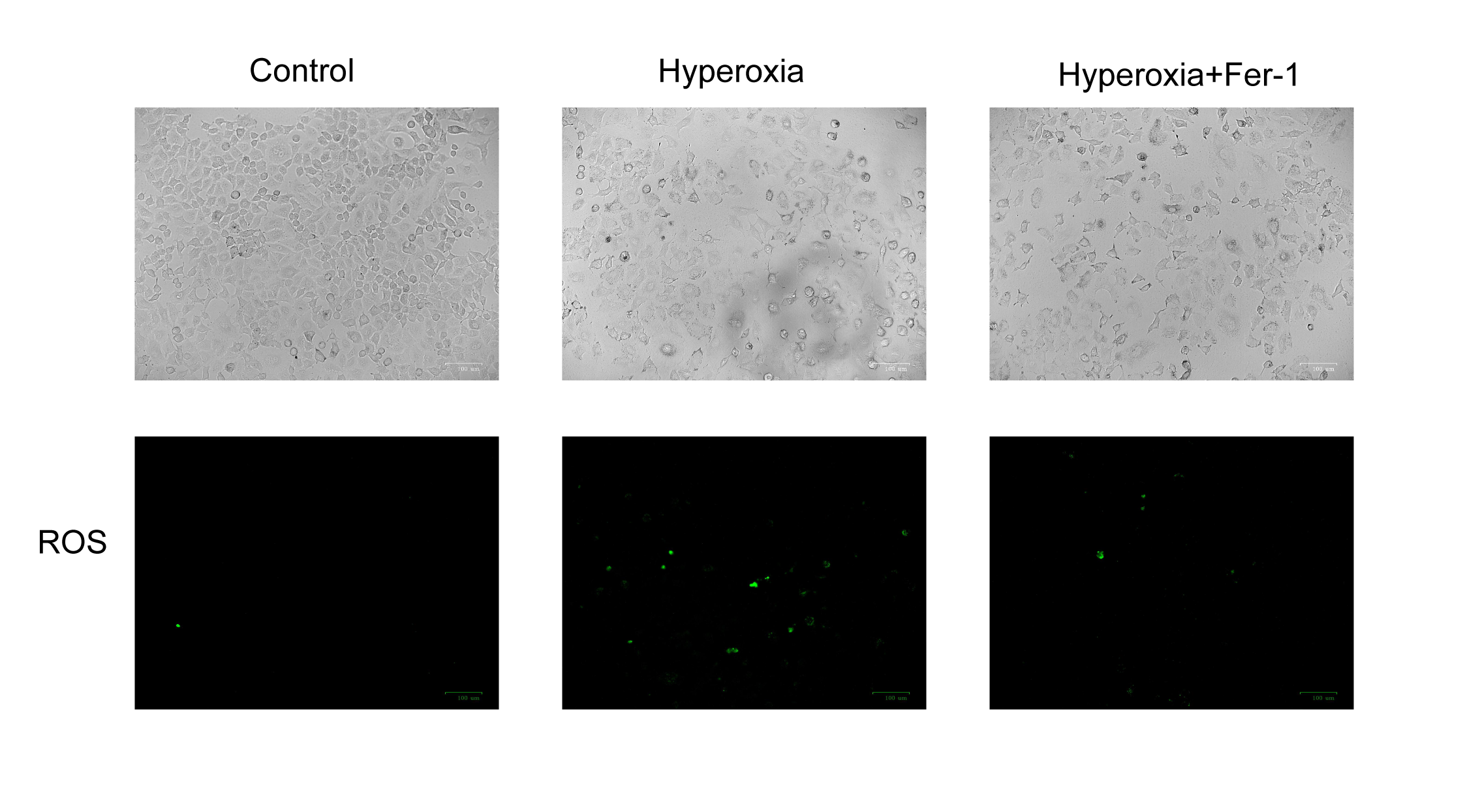

Supplement: S4 Fig — (TIF) [file pone.0291583.s004.tif]

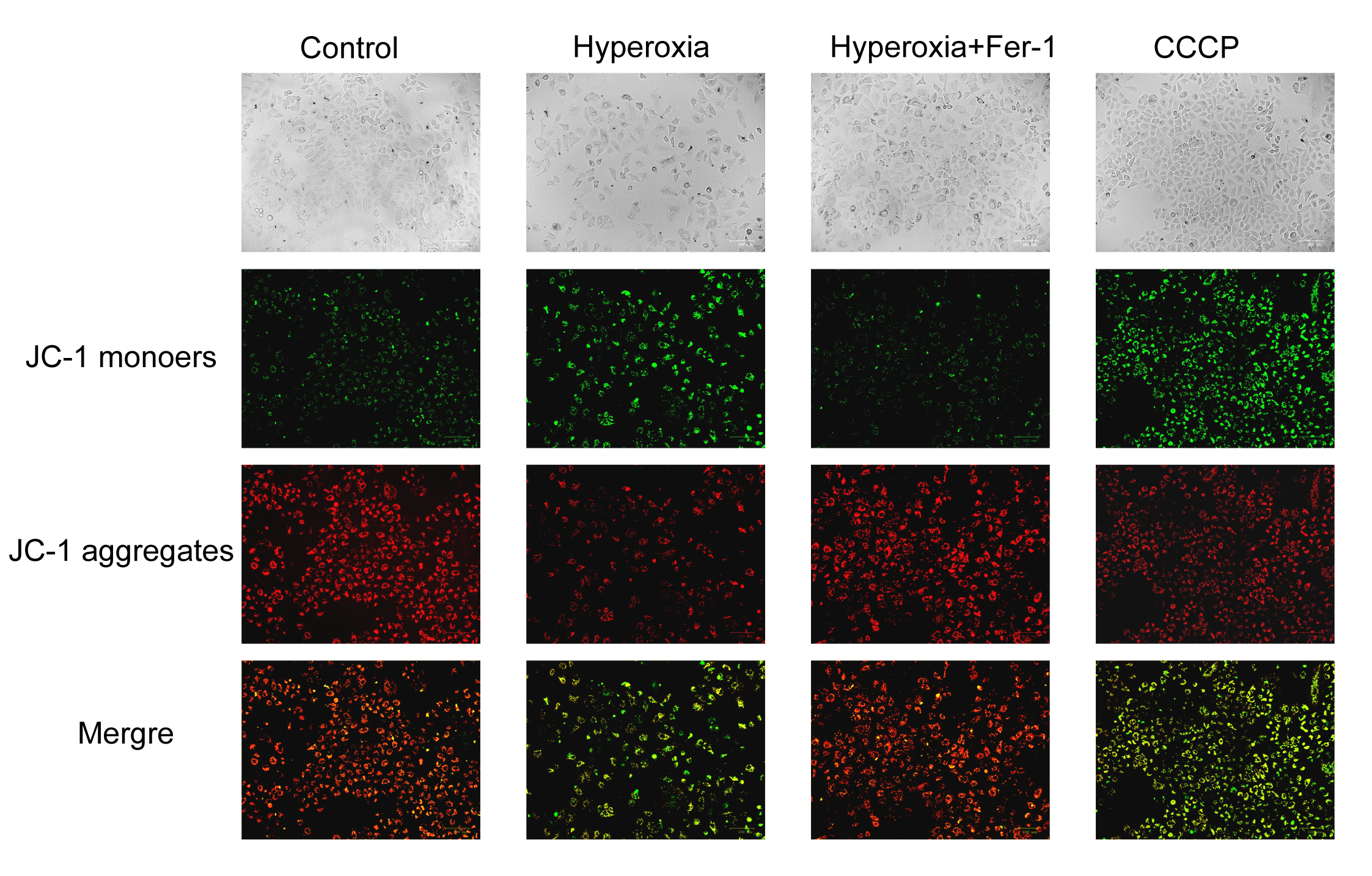

Supplement: S5 Fig — (TIF) [file pone.0291583.s005.tif]

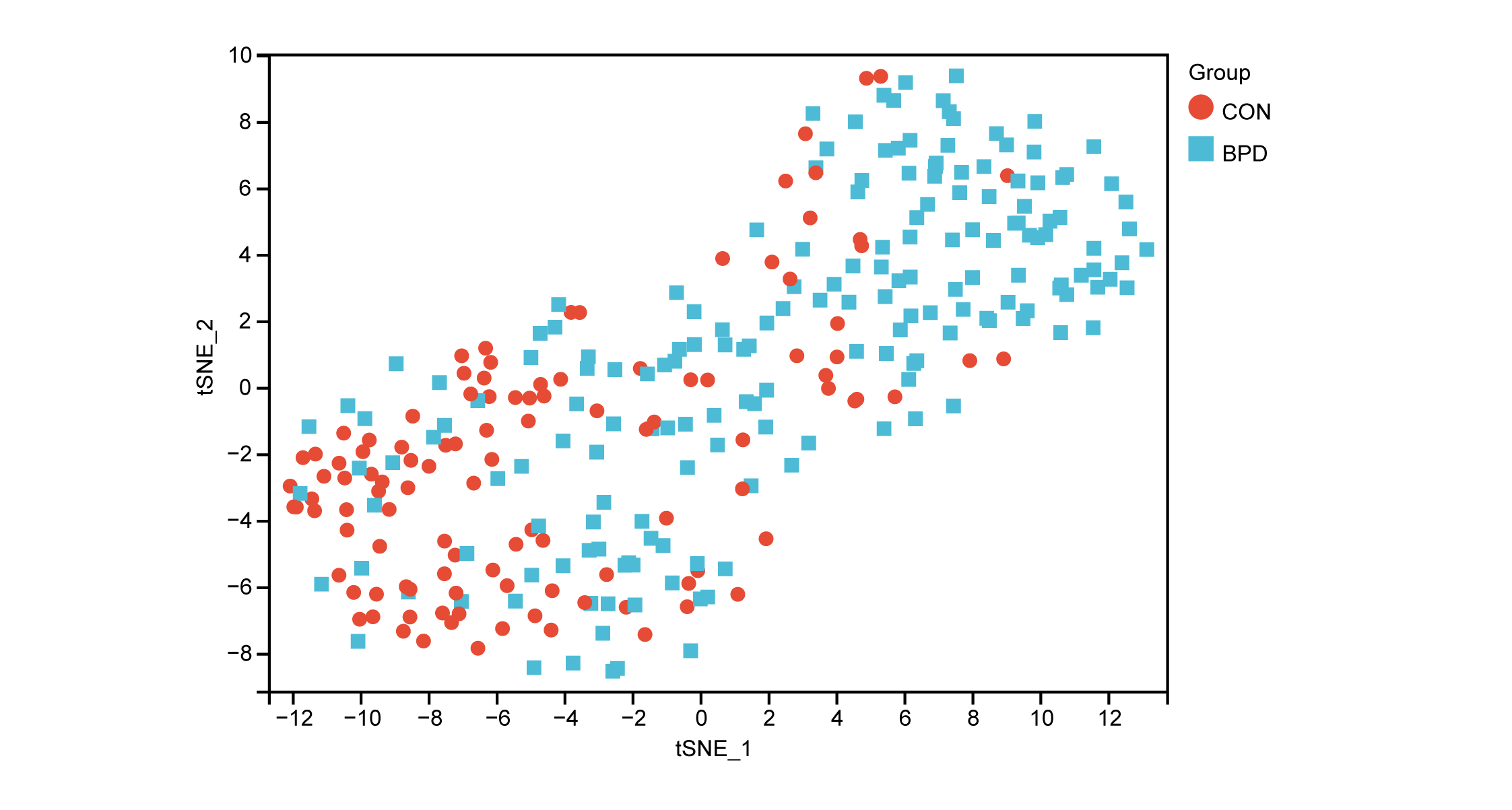

Supplement: S6 Fig — Blue points represent BPD group samples and red points represent CON group samples. (TIF) [file pone.0291583.s006.tif]

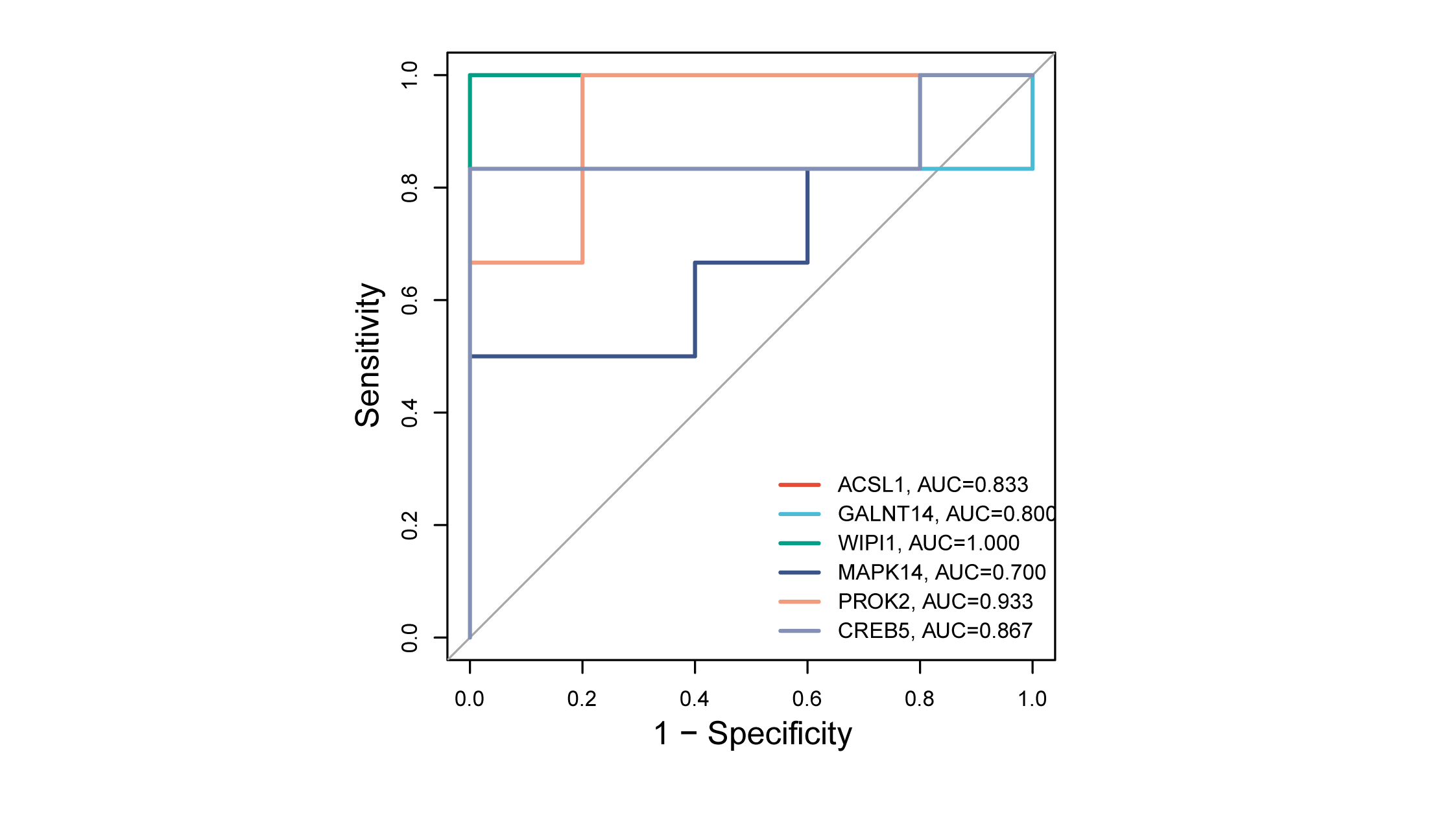

Supplement: S7 Fig — (TIF) [file pone.0291583.s007.tif]
